# Re-partitioning of Cu and Zn isotopes by modified protein expression

**DOI:** 10.1186/1467-4866-9-11

**Published:** 2008-10-10

**Authors:** Anette Büchl, Chris J Hawkesworth, K Vala Ragnarsdottir, David R Brown

**Affiliations:** 1 Department of Earth Sciences, University of Bristol, Wills Memorial Building, Bristol, BS8 4EU, UK; 2 Department of Biology and Biochemistry, University of Bath, Bath, BA2 7AY, UK; 3 School of Engineering and Natural Sciences, University of Iceland, Hjarðarhagi 6, 107 Reykjavík, Iceland

## Abstract

Cu and Zn have naturally occurring non radioactive isotopes, and their isotopic systematics in a biological context are poorly understood. In this study we used double focussing mass spectroscopy to determine the ratios for these isotopes for the first time in mouse brain. The Cu and Zn isotope ratios for four strains of wild-type mice showed no significant difference (δ^65^Cu -0.12 to -0.78 permil; δ^66^Zn -0.23 to -0.48 permil). We also looked at how altering the expression of a single copper binding protein, the prion protein (PrP), alters the isotope ratios. Both knockout and overexpression of PrP had no significant effect on the ratio of Cu isotopes. Mice brains expressing mutant PrP lacking the known metal binding domain have δ^65^Cu isotope values of on average 0.57 permil higher than wild-type mouse brains. This implies that loss of the copper binding domain of PrP increases the level of ^65^Cu in the brain. δ^66^Zn isotope values of the transgenic mouse brains are enriched for ^66^Zn to the wild-type mouse brains. Here we show for the first time that the expression of a single protein can alter the partitioning of metal isotopes in mouse brains. The results imply that the expression of the prion protein can alter cellular Cu isotope content.

## Background

Copper (Cu) and zinc (Zn) have essential roles in mammalian metabolism: copper in the formation of haemoglobin and red blood cells and Zn and Cu in several enzymes in a number of metabolic pathways. A number of neurodegenerative diseases are associated with abnormalities in the tissue distribution of these trace metals, such as Cu in prion disease [1,2], and Cu and Zn in Alzheimer's disease [3]. Cu has the two isotopes ^65^Cu and ^63^Cu, Zn has the five isotopes ^64^Zn, ^66^Zn, ^67^Zn, ^68^Zn and ^70^Zn. Different isotopes of the same element have different masses, which leads to different behaviour, and this contribution is concerned with the extent these isotopes are fractionated by small changes in a complex biological system, the brain.

Precise analyses of the ratios of transition stable isotopes has only been possible since the development of multi-collector inductively coupled plasma mass spectrometers and associated extraction techniques [4-7]. Variations in the isotopic composition are expressed by delta notation [δ^66^Zn = (^66^Zn/^64^Zn_sample_/^66^Zn/^64^Zn_standard _-1)*1000, and δ^65^Cu = (^65^Cu/^63^Cu_sample_/^65^Cu/^63^Cu_standard _-1)*1000 ], which is the deviation of a sample from an international standard in permil (1‰ = 0.1%). Variations in the isotopic composition of trace metals within organisms result from two effects. Biogeochemical processes in the environment lead to different isotopic compositions in, for example, soil, water, and plants. Isotope ratios may therefore be used to trace the origin, or source, of the element in question at the time it enters the body. Secondly, heavy stable isotope ratios fractionate during biochemical processes in organisms, and they are known to fractionate both during the uptake of trace metals into a cell, and as metals pass through membranes within the cell [8,9]. A study of Fe isotopes in human blood samples established that they were fractionated, and that the mean Fe isotope value is different in the blood of men and of women [10]. Such isotope fractionations reflect the fact that the isotope with a lower number of neutrons is kinetically more active and therefore used preferentially in biochemical processes.

This study focuses on the extent that small changes in a complex system affect trace metal Cu and Zn isotope ratios. If they do change such isotopes may be used as a new medical tool to investigate the pathways and partitioning of trace metals in human beings. Cu and Zn concentrations are routinely measured to determine their distribution in the brain, and the involvement of these elements in, for example, protein or enzyme function has been studied in detail [11,12]. Both metals are distributed throughout the brain with zinc concentrations being approximately twice that of copper (Cu being approximately 5 parts per million). Both metals are associated with large number of proteins and are important co-factors in the activity of many enzymes. Currently, no suitable method exists to examine pathways by which naturally occurring isotopes of metals are transported and partitioned within the animal body or whether they could be used to provide markers of abnormal transport and partitioning of trace metals in disease. Thus the question is the extent to which the isotope ratios of a metal are influenced by the expression of one particular protein in a biological system. Mice brains were analysed due to the availability of a suitable range of transgenically manipulated mice, allowing examination of the effects of alterations to a single proteins on the isotope ratios in a complex system like the brain.

The protein we chose to study was the prion protein, as it is a Cu-binding glycoprotein that can bind up to four Cu atoms, it is concentrated at synapses and may protect them from oxidative stress [13]. The metal ions (usually Cu) bind to a specific domain in the protein called the octameric repeat region. Deletion of this region from the protein abolishes metal binding to the protein [14]. Together with Cu the protein forms a complex that possesses anti-oxidant activity, and that may have important implications for synaptic homeostasis [13]. Prion protein misfolding is associated with the development of animal or human prion diseases (Scrapie, Bovine spongiform encephaloathy, Creutzfeldt-Jacob disease). Other trace metals such as Zn and Mn can substitute for Cu at the binding site [14]. However, while the affinity for Cu is high, that for Zn is very low. It is now well established that the prion protein (PrP) has an influence on cellular copper metabolism. As an example exposure of cells to high Cu concentrations increase PrP expression [15] and caused PrP to internalise [16], delivering copper into the cell [17]. Because PrP has a low zinc affinity it would be out competed by other zinc binding proteins and is unlikely to play any role in zinc metabolism.

This study presents for the first time accurate measurements of the ratio copper and zinc isotopes as they occur naturally in the brain. By studying these ratios in the brains of transgenic mice we sought to establish whether altered expression of a single protein can alter these isotopic ratios. We have shown that altered expression of the prion protein, in the normal cellular, non-aggregating isform, can selectively modify Cu isotope ratios. This implies that Cu isotopes are sensitive to the presence of different Cu-binding sites in the brain.

## Methods

### Mouse brains

The brains of different mouse strains were collected from adult (4 month old mice) or newborn mice. Mice studied included four lines of wild-type mice which included: MF1, 129Sv, FvB and C57BL/6 (Harlan). Also studied was a transgenic line included as a control for transgenic effects known as Harry [18] and three lines of mice in which the prion protein had been modified. The prion protein transgenic mice included prion protein knockout mice (PrP^o/o^ ) [19], mice overexpressing the protein (Tg20) [20] and mice which express a version of the prion protein lacking the octameric repeat region on a prion protein knockout background (C4) [21]. The comparable control mouse for the prion transgenic mice was the 129Sv mice.

### Isotope analyses

A method to separate Cu, Fe and Zn from silicate samples using a strongly basic anion resin has been described in detail by Marechal et al. (1999) [6]. This protocol, using AG MP-1 resin (Bio-Rad, CA, USA) was adopted here to accommodate smaller sample sizes and to decrease the size of the environmental blank contribution. It follows the approach of Archer and Vance [7] who provide more details of the analytical technique. The samples were digested using concentrated HNO_3 _+ H_2_O_2 _and concentrated HCl in a second step. Following sample digestion samples were loaded onto the column in 1 ml 7 M HCl + H_2_O_2_. The majority of matrix elements were removed by addition of a further 2 ml of 7 M HCl + H_2_O_2_, before collecting Cu in 8 ml 7 M HCl + H2O2; Fe was eluted by passing 4.5 ml 2 M HCl + H_2_O_2 _before finally collecting Zn in 4 ml 0.5 M HNO_3 _[7].

All analyses were performed on a ThermoFinnigan Neptune double focussing mass spectrometer at the University of Bristol (see [7] for a detailed description). Purified analyte fractions were introduced into the mass spectrometer in 2% HNO_3 _by means of a CETAC (Omaha, NE, USA) Aridus desolvating spray chamber fitted with a CPI (Amsterdam, Neth.) PFA nebuliser and spray chamber to give enhanced sensitivity. Instrumental mass fractionation was corrected for using external normalisation techniques described by Marechal et al. [6], with careful attention being paid to matrix matching of samples and standards (cf. Archer and Vance [7]). We used the Cu standard from NIST and the Zn standard from Lyons JMC, and ^60^Ni was monitored to correct for the isobaric interference of ^64^Ni on ^64^Zn.

Statistical analysis of the data was carried out using "Analysis of Variance" (ANOVA) or with a two tailed Student's t-test.

## Results and discussion

**Figure 1:**
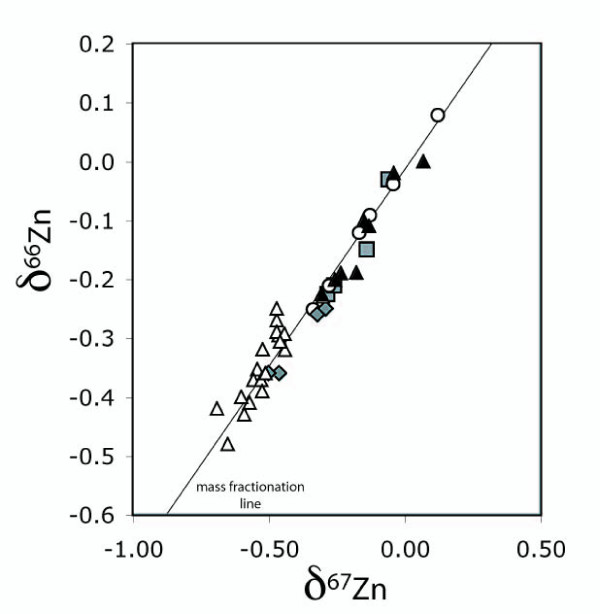
**A plot of δ^66^Zn versus δ^67^Zn for all the brains analysed (Table 1) showing that the observed shifts in isotopic ratios lie on the mass fractionation line), and thus they all obey the mass fractionation law.** Open triangles – wild mouse strains; filled triangles – prion protein depleted; open circles – TG20; grey squares – C4; grey diamonds – line Harry.

Cu and Zn isotopes were analysed by MC-ICP-MS and the results are presented in Table 1. The internal reproducibility for measurement of Cu and Zn isotopes was 0.03‰ (2 sigma). The external reproducibility for Cu and Zn by sample bracketing was 0.08‰ (2 sigma) and for Zn with a double spike 0.04‰ (2 sigma). All the observed shifts in isotopic ratios lie on the mass fractionation line (Fig. 1), and thus they all obey the mass fractionation law.

**Table 1:** Cu and Zn isotopic compositions of the mouse brains

	delta^65^Cu/^63^Cu	delta^66^Zn/^64^Zn	delta^67^Zn/^64^Zn
MF1	-0.59	-0.27	-0.47
	-0.35	-0.30	-0.46
	-0.33	-0.35	-0.54
	-0.49	-0.29	-0.47
	-0.43	-0.23	-0.30
	-0.47	-0.29	-0.44

129	-0.29	-0.25	-0.47
	-0.23	-0.42	-0.69
	-0.78	-0.37	-0.56
	-0.77	-0.32	-0.52

Fvb	-0.12	-0.37	-0.53
	-0.16	-0.39	-0.52
	-0.38	-0.32	-0.44
	-0.3	-0.36	-0.51

C57	-0.35	-0.31	-0.46
	-0.46	-0.41	-0.57
	-0.7	-0.43	-0.59
	-0.73	-0.48	-0.65
	-0.42	-0.40	-0.60

PrP-KO (adult)	-0.56	-0.20	-0.26
	-0.16	-0.19	-0.23
	-0.14	-0.11	-0.13
	-0.16	-0.02	-0.04

PrP-KO (newborn)	-0.33	-0.23	-0.30
	-0.17	-0.19	-0.18
	-0.1	0.00	0.07
	-0.38	-0.10	-0.15

C4	-0.02	-0.15	-0.14
	0.31	-0.22	-0.29
	0.04	-0.21	-0.26
	-0.09	-0.03	-0.06

TG20	0.18	0.08	0.12
	-0.36	-0.04	-0.05
	0.26	-0.09	-0.13
	-0.44	-0.12	-0.17
	-0.37	-0.25	-0.34
	-0.58	-0.21	-0.28

Line Harry	-0.14	-0.26	-0.32
	-0.17	-0.36	-0.46
	-0.21	-0.25	-0.29
	-0.46	-0.36	-0.5

The Cu and Zn isotope ratios of mice brains, expressed in the delta notation (as discussed in the text. The different samples are: MF1, 129, FvB and C57 BL/6 – wild-type mouse brains; PrP-KO (adult) prion protein deleted mouse brains, adult; PrP-KO (newborn) prion protein deleted mouse brains, new born; C4 – Cu binding domain of the prion protein deleted; TG20 – prion protein overexpressed; Line Harry – transgenic mouse brains with the genetic changes have no connection with Cu or Zn.

In order to eliminate age or diet effects, only mice held under controlled conditions were chosen. The mice were 4 months old and they had all been fed with the same mouse pellets. Cu and Zn isotopes were analysed in 'normal' wild type mouse brains from 4 different strains with no genetic modifications (n = 19), in prion protein overexpressed mouse brains (TG20, for details see [20]) (n = 6), in brains in which the prion protein had been deleted (PrP^o/o^, for details see [19]) (n = 4), in brains in which the copper-binding region of the prion protein had been knocked out (C4, for details see [21]) (n = 4) and in other transgenic mice from the line Harry which carry a luciferase transgene driven by promoter/enhancer elements from the Igf2/H19 locus (for details see [18]) (n = 4). Four new born mice brains in which the prion protein had been deleted were also analysed to assess the influence of age on the Cu and Zn isotopic composition. In all cases whole brains were dissolved in concentrated nitric and hydrochloric acid for analysis.

**Figure 2:**
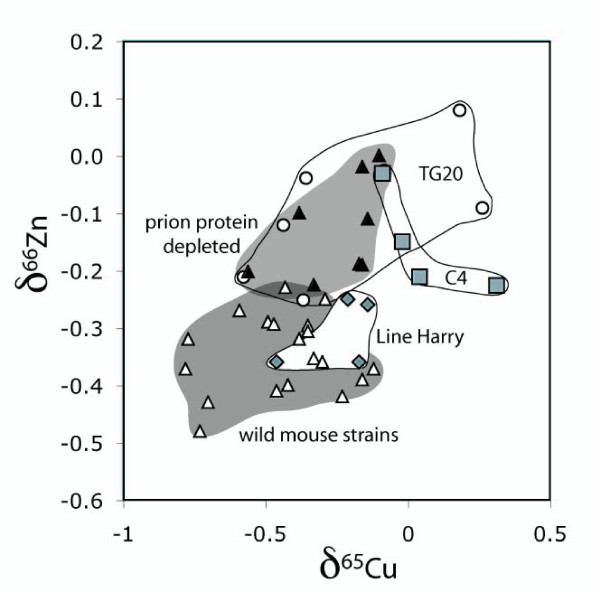
**A plot of δ^65^Cu and δ^66^Zn for all the mice brains analysed.** The wild-type mouse brains are from four different strains, including MF1 (n = 6), 129 SV (n = 4), C57BL6 (n = 5), FVB (n = 4), and the transgenic mouse brains are from the line Harry (n = 4) in which the genetic changes have no connection with Cu or Zn. The C4 transgenic mouse brains have the Cu binding domain of the prion protein deleted (C4, n = 4), PrP have the prion protein deleted (n = 4) and TG20 have the prion protein overexpressed (n = 6).

The δ^65^Cu values in the wild type mice brains of four different strains (MF1, 129, C57 BL/6 and FvB) varied between -0.12 and -0.78 permil, and δ^66^Zn between -0.23 and -0.48 permil (the results are plotted on a diagram of δ^65^Cu against δ^66^Zn in Figure 2). The range of Cu and Zn isotopic ratios in the wild-type mice brains is not understood, but there was no significant difference between the isotope ratios of the different wild type mice strains. In more detail the two sample t-test, indicates that for most of the samples the wild-type mice brains show no significant differences to each other (e.g. 129 – C57: δ^65^Cu: t = 0.09, df = 7, p = 0.92, δ^66^Zn: t = 1.47, df = 7, p = 0.18; δ^67^Zn: t = 0.25, df = 7, p = 0.80). This implies that the different genetic backgrounds had no significant influence on the isotopic ratios. These results present the first assessment of Cu and Zn isotope ratios in the brain.

There has been one other study on the fractionation of Cu and Zn isotopes in biological tissue. Cu uptake in vitro into the apoprotein of azurin expressed in Escherichia coli fractionates the Cu isotopes, and the azurin (δ^65^Cu -1.64) was enriched in the lighter isotope relative to the source material (δ^65^Cu -0.11) [9]. In an in vivo experiment, where the intact Cu protein azurin was synthesised directly inside cells of the bacterium Pseudomonas aeruginosa, the Cu isotope composition of the azurin (δ^65^Cu -1.09) was also enriched in the lighter isotope [9]. It appears that the Cu isotopic ratios of the mouse brains lie within the range of other biological samples.

In order to determine if any form of genetic manipulation could alter isotope ratios a transgenic line was chosen at random and the isotope ratios in the brain compared to that for the brains of the matching mouse strain from which they were derived (C57BL/6). The δ^65^Cu and δ^66^Zn values of the wild type mice brains overlap with those from transgenic mouse brains from the line Harry in which the genetic changes have no connection with Cu or Zn (Fig. 2). These results show that transgenic manipulation on its own did not change the Cu or Zn isotopic composition significantly.

We then studied a series of different transgenic mice in which transgenic manipulation of a single protein is known to alter the copper content of the brain. The prion protein in these mice was either either overexpressed (Tg20) deleted (PrP^o/o^) or modified to lack the main metal binding domain (C4). All three groups plot in different fields to the wild type and line Harry mice brains on the plot of Cu versus Zn isotopes (Fig. 2). Specifically the prion protein overexpressed (Tg20) deleted (PrP^o/o^) or modified to lack the main metal binding domain (C4) brains all have more positive Zn isotope ratios than the wild type and line Harry mice brains (δ^66^Zn: R^2 ^= 0.70, p < 0.001; δ^67^Zn: R^2 ^= 0.76, p < 0.001). In contrast, only the C4 samples, those without the Cu binding domain of the prion protein, have consistently more positive Cu isotope ratios (Fig. 2), as do two samples from TG20. The average δ^65^Cu value of the C4 brains is 0.57 permil enriched in the heavy Cu isotope (^65^Cu) compared to the wild type mice brains, and this difference was highly significant (ANOVA, R^2 ^= 0.45, p = 0.01). Analysis was also carried out for these results with the Student's t-test. The analyses confirm the inferences from the figures, namely, that 129 – C4 are significantly different (δ^65^Cu: t = -3.339, df = 6, p = 0.02; δ^66^Zn: t = -3.269, df = 5, p = 0.02; δ^67^Zn: t = -5.004, df = 5, p = 0.01) and that 129 – PrP^o/o ^show no significant differences for the Cu isotope ratios (δ^65^Cu: t = -1.454, df = 6, p = 0.19), but are different for the Zn isotope ratios (δ^66^Zn: t = -3.792, df = 6, p = 0.01; δ^67^Zn: t = -5.746, df = 6, p = 0.001)).

Finally, we tested whether changes in isotopes could be something that occurs with increased age. We examined the metal isotope ratios in new born mice and compared them to those from the adults in this study. The one-day old mice brains with the prion protein deleted (δ^65^Cu ranges between -0.10 and -0.38, and δ^66^Zn between 0.00 and -0.23 permil) fell within the field of the older mice brains in which the prion protein had been deleted (δ^65^Cu ranges between -0.14 and -0.56 permil, and δ^66^Zn between -0.02 and -0.20 permil, Table 1). Comparison between PrP^o/o^-adult and PrP^o/o^-young using the Student's t-test confirm that these two groups show no significant differences from each other (δ^65^Cu: t = -0.082, d = 6, p = 0.98; δ^66^Zn: t = 0.00, df = 6, p = 1.00; δ^67^Zn: t = -0.272, df = 6, p = 0.80). This strongly suggests that changes with age did not result in analytically significant differences in Cu and Zn isotopic ratios, at least in the first 4 months.

Neither overexpression nor the lack of expression of PrP alters the partition of the Cu isotopes. However, expression of a form of PrP that cannot bind Cu results in increased levels of the heavier Cu isotope. The implication is that the presence of PrP inhibits mechanisms that would otherwise regulate the Cu content to maintain the normal Cu isotope ratio. When PrP can bind Cu these alternative mechanisms are not required. When PrP is not expressed the alternative mechanisms are activated and able to compensate for the loss of expression. At present, if these alternative balancing mechanisms exist, they remain unknown. Tg20 mice express 10 time the normal level of PrP with little measurable variation in the level of the protein times the normal levels of PrP [20]. However, previous studies have shown the amount of Cu bond per molecule of PrP in Tg20 mice was greatly reduced when compared to wild-type [22]. This implies that the impact of increased expression of PrP on Cu isotopes is limited by the availability of Cu to bind to it. However, it is important to note the huge variation in the values for the TG20 mice. This variation is greater than between different wild-type mouse lines and suggests that overexpression of PrP causes disturbance to maintenance of isotope ratios at an individual level. This would imply that expression of PrP is not the prime mechanism by which a cell maintains the ratio balance.

A very interesting observation is that wild-type mice show little variation in the ratios of the three Zn isotopes (Fig. 1). This again implies tight regulation of Zn isotope content of the brain. However, any form of manipulation of PrP resulted in a significant change to the ratios. It is possible that either loss or overexpression of a copper binding protein could have a similar effect on the way a cell processes Zn. In the case of deleting PrP it is possible that compensatory mechanisms, increasing Cu uptake or utilisation could be non selective and also alter Zn uptake. In the case of overexpression of PrP, PrP itself could alter internalisation of Zn either by directly binding Zn as has been suggested [14] or indirectly increasing transport of Zn. In the latter case other non selective transport proteins such as divalent metal transporter (DMT-1) could show an increase transport of Zn due to decreased availability of Cu (due to it being bound to PrP). Again, the mice overexpressing PrP showed high variability in Zn isotopic ratios in the brain. This variation was similar to that seen for Cu ratios. Although there is no information concerning the mechanism, it does suggest that there is some form of co-regulation of this process maintaining isotope ratios for different metals. At the level of individuals the greater variation in values implies an increased complexity of the alternative routes for metal entry into cells.

In terms of the relationship of Zn isotopes ratios to PrP, the simples explanation of the results is that they are unrelated to the metal binding capacity of PrP. While changes in Cu isotopes can clearly be related to the loss of metal binding to the protein, the change in Zn isotopes is only consistent with a genetic modification of the mice involving the PrP gene. This might suggest that Zn isotope ratios are more sensitive to the modification of cellular metabolism that Cu isotope ratios. This means that the study of Cu isotope ratios is more likely to uncover mechanisms that could be utilised to understand how a protein alters copper metabolism than the study of Zn isotope ratios. This possibly relates to the different nature of the two metals and the greater role of Cu in reactions that can cause damage to cells through oxidative stress.

## Conclusion

This study establishes for the first time that a minor change to a biological system can alter the distribution of metal isotopes in ways that could not be predicted. Until this study it has not been possible to establish the wild-type ratios of Cu and Zn isotopes. The observed differences in the Cu and Zn isotope composition of mouse brains with different protein expression profiles supports the hypothesis that trace metal isotopes can be used to examine processes leading to brain damage and disease and pathways by which metals are transported through the animal body. As the altered ratios were not observed in the Harry transgenic mouse line it is clear that these changes are specific for relevant metal binding proteins. These observations may open up a new field of medical research using geochemical tools. Other applications may include other neurodegenerative diseases that are related to trace metals like Alzheimer's disease [3,23,24] or Parkinson's disease [25]. However, further studies of the basic mechanisms involved are essential before such information could have any meaning in a diagnostic sense. Nevertheless, these findings highlight that an essential unexplored aspect of metalochemistry has a significant biological relevance. How different metal isotopes are utilised biologically remains unknown but our data provides evidence that they are used differently. If cells in the brain selectively regulate the isotopes that enter the brain, then differential utilisation of these isotopes by the brain might lead to important consequences for important cellular processes. The consequences of altering this differential utilisation might have a significant impact on health issues.

## Authors' contributions

AB carried out the experimental procedures and assisted with manuscript preparation. DRB provided mouse brains and background for biological aspects of the project and contributed to experimental planning and manuscript preparation. CJH and KVR were responsible for supervision, project planning and preparation of the manuscript.
